# Aberrant gene expression of superoxide dismutases in *Chlamydia trachomatis*-infected recurrent spontaneous aborters

**DOI:** 10.1038/s41598-022-18941-y

**Published:** 2022-08-29

**Authors:** Ankita Ray, Tanu Bhati, Dibyabhaba Pradhan, Renu Arora, Suhel Parvez, Sangita Rastogi

**Affiliations:** 1 Molecular Microbiology Laboratory, ICMR-National Institute of Pathology, Sriramachari Bhawan, Safdarjung Hospital Campus, Post Box no. 4909, New Delhi, 110029 India; 2 ICMR Computational Genomics Centre, All India Institute of Medical Sciences, Indian Council of Medical Research, New Delhi, 110029 India; 3 Department of Obstetrics and Gynecology, Vardhman Mahavir Medical College (VMMC) and Safdarjung Hospital, New Delhi, 110029 India; 4 Department of Medical Elementology and Toxicology, Jamia Hamdard, New Delhi, 110062 India

**Keywords:** Medical research, Pathogenesis

## Abstract

Study aimed to characterize the expression of antioxidant genes SOD1 and SOD2 in *Chlamydia trachomatis*-induced recurrent spontaneous aborters and further determine their role by in silico analysis. First void urine was collected from 130 non-pregnant women with history of recurrent spontaneous abortion (RSA) (Group I) and 130 non-pregnant women (Group II; control) attending Obstetrics and Gynecology Department, SJH, New Delhi, India. *C. trachomatis* detection was performed by conventional PCR in urine. Gene expression of SOD1 and SOD2 was performed by quantitative real-time PCR. Further, its interacting partners were studied by in silico analysis. 22 patients were positive for *C. trachomatis* in Group I. Significant upregulation was observed for SOD2 gene in *C. trachomatis-*infected RSA patients while SOD1 was found to be downregulated. Increased concentration of oxidative stress biomarkers 8-hydroxyguanosine and 8-isoprostane was found in *C. trachomatis-*infected RSA patients. Protein–protein interaction (PPI) of SOD proteins and its interacting partners viz.; CCS, GPX1, GPX2, GPX3, GPX4, GPX5, GPX7, GPX8, CAT, PRDX1, TXN, SIRT3, FOXO3, and AKT1 were found to be involved in MAPK, p53 and foxo signaling pathways. Molecular pathways involved in association with SODs indicate reactive oxygen species (ROS) detoxification, apoptotic pathways and cell cycle regulation. Overall data revealed alleviated levels of SOD2 gene and decreased expression of SOD1 gene in response to *C. trachomatis-*infection leading to production of oxidative stress and RSA.

## Introduction

*Chlamydia trachomatis* infection is the most common and frequently reported sexually transmitted disease and an important etiology behind adverse pregnancy outcomes. The involvement of the pathogen in pregnancy associated complications is still under active investigation. Recent studies have established probable association of *C. trachomatis* with RSA but limited reports are available describing the mechanism underlying *C. trachomatis*-induced RSA^1,2^. The pathogen induces early pregnancy failure by causing trophoblast infiltration leading to production of cytokines and ROS from macrophages and polymorph nuclear leukocytes intervening embryo implantation due to inflammatory response of the infected cell^3,4^. Genital tract infection by *C. trachomatis* can lead to augmented production of free radicals and thereby oxidative stress (OS) influencing the pathophysiology of pregnancy related complications. Pathological response to *C. trachomatis* infection leading to production of ROS and lipid peroxidation and finally cell death and inflammation releases the infectious elementary bodies to distant sites spreading the infection^5,6^ thus affecting reproductive functions.

Several studies have been carried out which have established the role of OS in RSA and other obstetric ailments^7–9^. Placental oxidation during pregnancy is known to be one of the important causes behind RSA^10^. Imbalance in the ratio of antioxidants and oxidants has been an important cause behind RSA^7,11^. However, *C. trachomatis* associated RSA molecular pathways is unclear and further research is required. OS occurs when the level of oxidants increases than the level of antioxidants leading to the generation of ROS. Certain level of ROS is required for various physiological functions and defense against pathogenic infections but overproduction of the same can be detrimental for cell survival causing significant oxidation of DNA, inhibition of protein synthesis and lipid peroxidation^12^. The various phases of pregnancy beginning from conception to delivery can undergo abnormal events due to varied levels of OS^13^. ROS generation affects trophoblast migration, proliferation and apoptosis^14^. High levels of ROS are known to impair placental development and trophoblast degeneration by inducing cell apoptosis and thus affecting reproductive functions. Role of antioxidants has been well studied in pregnancy and various obstetric conditions such as RSA.

SODs are a class of antioxidant genes which catalyze the conversion of superoxide to oxygen and hydrogen peroxide. They are the first line of defense against microorganisms and are expressed by all aerobic organisms. They control the release of ROS and signaling functions taking place in cellular life. SODs such as MnSOD and Cu–Zn SOD are oxidoreductase enzymes localized in the mitochondria and cytoplasm respectively responsible for dismutation of superoxide radicals to molecular oxygen. Change in expression of SODs has been implicated in certain pathological conditions and diseases such as cardiovascular, neurogenetic disorders^15^. In a study carried out in RSA patients, decreased levels of SOD was found as compared to pregnant women^11^. In another study, decreased levels of antioxidants GPX, catalase and SOD was found in serum of RSA patients when compared to control group^16^.

Studies also reveal that the survival of pathogens is determined by the redox system of the host. It survives by inducing OS and further chronic conditions are established by reduced ROS levels^17^. *C. trachomatis* is known to target NADPH oxidase to shut down the production of ROS produced by the host during pathogen infection^18^. Cell line studies of macrophages infected with with *C. pneumoniae* infection indicated increased activity of SOD and GPX^19^. Another study evaluated the ROS and Reactive Nitrogen Species (RNS) production in monocytes infected by *C. trachomatis* and found that the infection was cleared from the monocytes within a period of 06 h due to release of ROS and RNS^20^. In a study by Zaki and coworkers, oxidative biomarkers such as nitrite and reduced glutathione were increased in spontaneous abortion patients serologically positive for *C. trachomatis* as compared to *C. trachomatis*-negative patients^21^. Oxidative damage to DNA as a result of infection leads to increase in OS biomarkers such as 8-hydroxy-2-deoxyguanosine (8-OHdG) which has been reported to increase in *C. trachomatis* infection in women with tubal infertility causing oxidative damage to DNA^22^.

Despite the availability of literature confirming to the role of SOD genes in RSA, the pathophysiology of *C. trachomatis* in generating OS in RSA is poorly understood; therefore this study was conducted to determine the relative expression of SOD1 and SOD2 genes and concentration of OS biomarkers 8-OHdG and 8-isoprostane in *C. trachomatis*-induced RSA patients and analyze interacting partners and functions of SOD1 and SOD2 genes by STRING database. GEO microarray datasets were obtained to determine their interaction if any, with superoxide dismutase genes.

## Results

### Clinical characteristics of enrolled patients

The mean (± SD) age of patients in Group I and II were 28.72 ± 4.32 and 27.72 ± 3.34, respectively. All enrolled patients were of Indian origin and age-matched. The clinical characteristics of RSA and control patients are summarized in Table 1.

**Table 1:** Clinical characteristics of aborters and control patients.

Clinical characteristics	Range	RSA Group (n = 130) (%)	Control (n = 130) (%)	P value
Age (years)	< 20	33 (25.38)	31 (23.84)	0.735*
	20–25	26 (20)	28 (21.53)	
	26–30	34 (26.15)	41 (31.5)	
	31–35	37 (28.46)	30 (23.07)	
		Mean age = 28.72 ± 4.32	Mean age = 27.72 ± 3.34	
Gravidity	1	0	0	0.5162*
	2	0	86	
	> 2	130 (100)	44	
		Average gravidity = 3		
Parity	0–1	0	0	NS
	2 → 2	0	130	
No. of abortions	0–1	0	0	NS
	1–2	0	0	
	3 → 3	130	0	

*RSA* Recurrent spontaneous abortion, *NS* non-significant.

*Statistically non-significant.

### *Chlamydia trachomatis* detection in urine

130 women with history of two or more RSA comprised Group I. 17% RSA patients (n = 22) were found to be infected with *C. trachomatis* using MOMP and plasmid primers. Group I was further sub- divided into Group Ia (*C. trachomatis*- infected RSA patients; n = 22) and Group Ib (*C. trachomatis*-infected RSA patients; n = 108) on the basis of presence/ absence of *C. trachomatis.* No positivity for *C. trachomatis* was found in control patients (Group II; n = 130).

### Quantitative detection of *Chlamydia trachomatis* by real-time PCR

**Figure 1:**
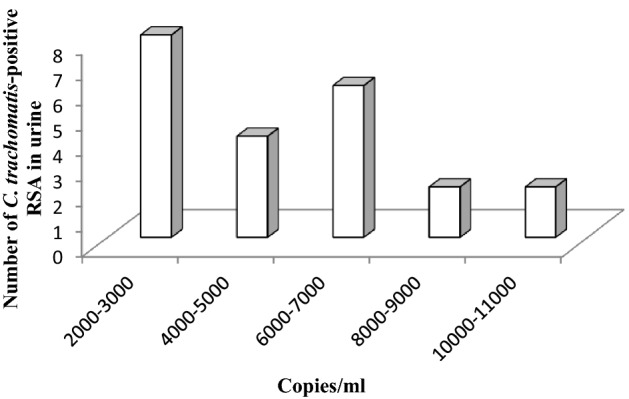
Distribution of *Chlamydia trachomatis* DNA load in urine samples of Group I patients by MOMP gene qRT-PCR. *RSA* Recurrent spontaneous aborters.

Real-time PCR was performed by the SYBR green-based chemistry to quantitate the *C. trachomatis* copies in urine of groups I–II patients. Standard curve was plotted by preparing serial dilutions using the known concentrations of *C. trachomatis-*positive control. A total of 2000–11,000 copies/ml were detected in the urine of *C. trachomatis-*positive RSA (Fig. 1). 08, 04, 06, 02, 02 patients had a copy number of 2000–11,000, 4000–5000, 6000–7000, 8000–9000, 10,000–11,000/ml respectively.

### Concentration of urine 8-OHdG and 8-isoprostane in recurrent spontaneous aborters

Mean urine 8-OHdG level was estimated in controls and *C. trachomatis*-positive as well as uninfected RSA and it was found that the 8-OHdG concentration was significantly high (99.65 ng/ml) in the *C. trachomatis* positive RSA, as compared with both uninfected RSA (50.54 ng/ml) and the control group (31.97 ng/ml; Mann–Whitney test, ‘p’ < 0.05).

**Figure 2:**
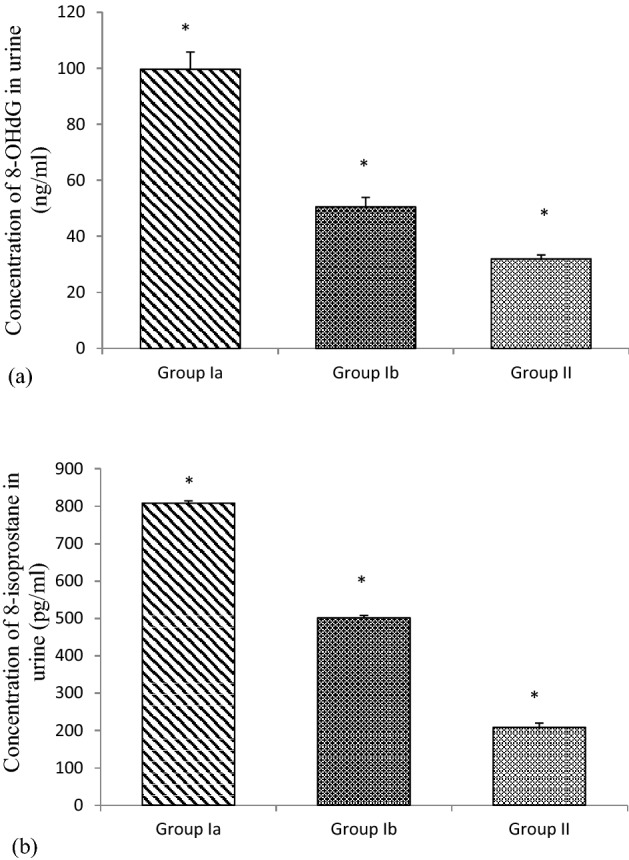
Concentration of 8-deoxyhydroxyguanosine (ng/ml) and 8-isoprostane (pg/ml) in urine of RSA and controls. *Group Ia* *C. trachomatis*-positive recurrent spontaneous aborters, *Group Ib* *C. trachomatis*-negative recurrent spontaneous aborters, *Group II* *C. trachomatis*-negative non-pregnant women with ≥ 2 successful deliveries (Controls); *indicates ‘p’ value < 0.05.

Mean urine 8-isoprostane concentration was significantly high (808.28 pg/ml) in the *C. trachomati*s-positive RSA, as compared with both uninfected RSA (501.39 pg/ml) and control group (208.02 pg/ml; Mann–Whitney test, ‘p’ < 0.05) (Fig. 2).

### Quantitative expression of SOD1 and SOD2 genes

**Figure 3:**
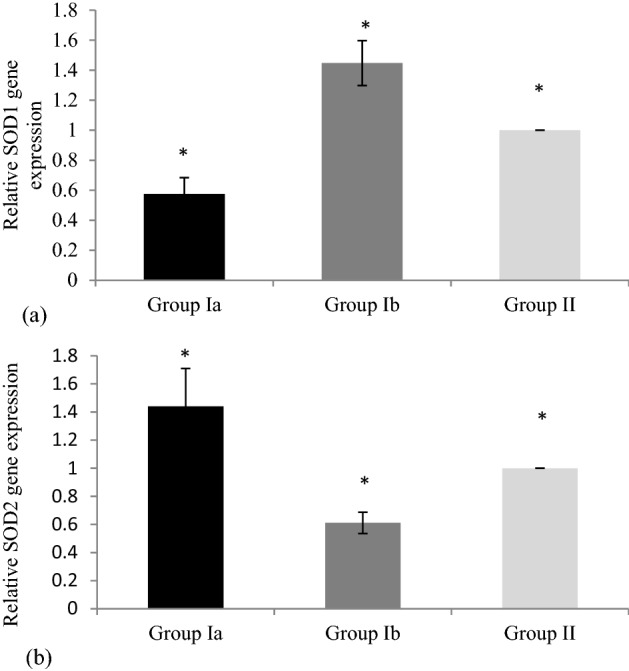
Relative expression of (**a**) SOD1 and (**b**) SOD2 genes in urine of *Chlamydia trachomatis*-positive recurrent spontaneous aborters. *Group Ia* *C. trachomatis*-positive recurrent spontaneous aborters, *Group Ib* *C. trachomatis*-negative recurrent spontaneous aborters, *Group II* *C. trachomatis*-negative non-pregnant women with > 2 successful deliveries (Controls).

To determine whether *C. trachomatis* affects the expression of SODs at the transcript level in RSA patients, q-PCR was performed and the expression of Group Ia was compared with Group II. Constitutively expressed gene GAPDH was used as the endogenous control. Analysis revealed significant downregulated mRNA expression with a relative fold change of 0.52 for SOD1 in Group Ia (n = 22) as compared to Group II (n = 130) while fold change of 1.4 was observed in Group Ib (n = 108) as compared to Group II (n = 130) (Mann–Whitney non- parametric test; p < 0.05). In case of SOD2, analysis revealed significant upregulated mRNA expression with a fold change of 1.4 in Group Ia (n = 22) *versus* Group II (n = 130) whereas a fold change of 0.69 was found in Group Ib (n = 208) *versus* Group II (n = 130) (Mann–Whitney test-non parametric test; p < 0.05). (Fig. 3).

### Correlation between genes SOD1 and SOD2 and gestational age (GA)

**Figure 4:**
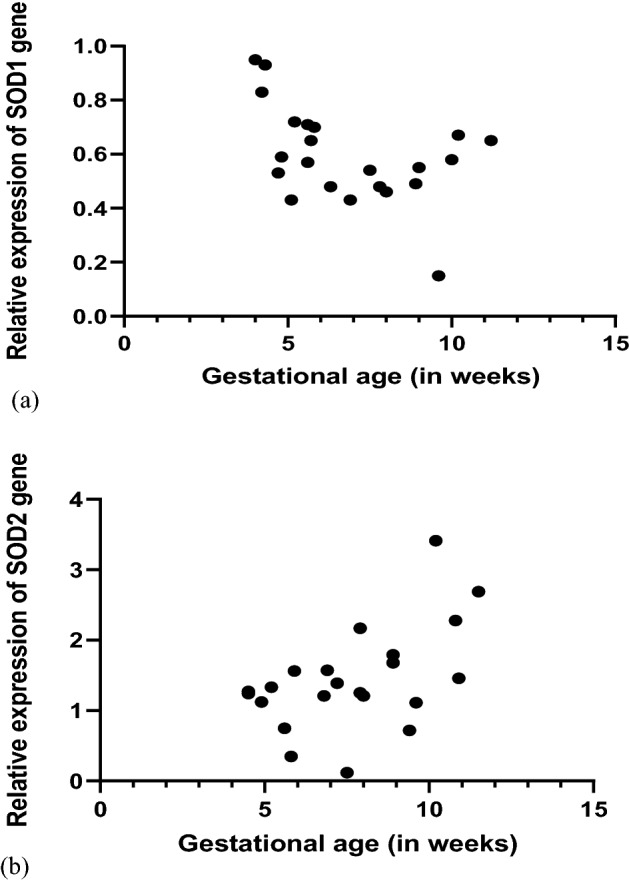
Determination of correlation between relative expressions of (**a**) SOD1 (**b**) SOD2 with gestational age in *Chlamydia trachomatis*-positive recurrent spontaneous aborters (Spearman’s rank correlation test).

SOD1 and SOD2 gene expression was correlated with GA. Positive significant correlation was observed between SOD2 and GA (r = 0.449, p = 0.038) while a negative correlation was observed between SOD1 and GA (r = − 0.462, p = 0.047) (Fig. 4).

### Correlation between *C. trachomatis* load and SODs

**Figure 5:**
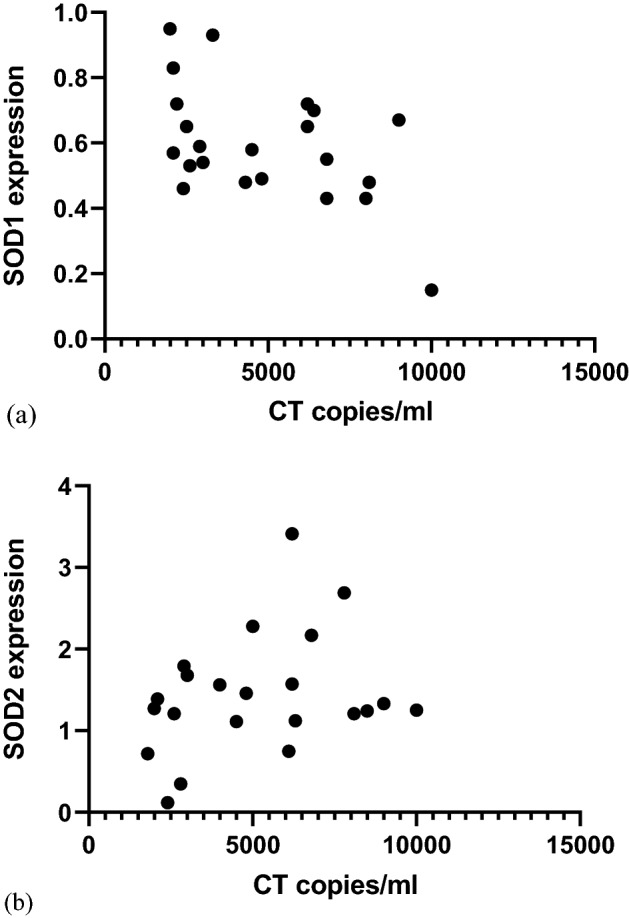
Determination of correlation between *Chlamydia trachomatis* copy load (**a**) SOD1 (**b**) SOD2 gene expression in *C. trachomatis*-positive recurrent spontaneous aborters (Spearman rank correlation test). *CT* *Chlamydia trachomatis.*

The expression of SOD1 and SOD2 were correlated with the chlamydial load in the urine of infected RSA. A statistically significant positive correlation was observed between SOD2 and *C. trachomatis* copy load (r = 0.468; ‘p’ = 0.027). Chlamydial load and SOD1 gene was found to be negatively correlated in *C. trachomatis*-positive RSA (r = − 0.45, ‘p’ = 0.035) indicating that a greater *C. trachomatis* copy load leads to decreased expression of SOD1 gene in RSA patients (Fig. 5).

### Analysis of PPI network with RSA DEGs

**Figure 6:**
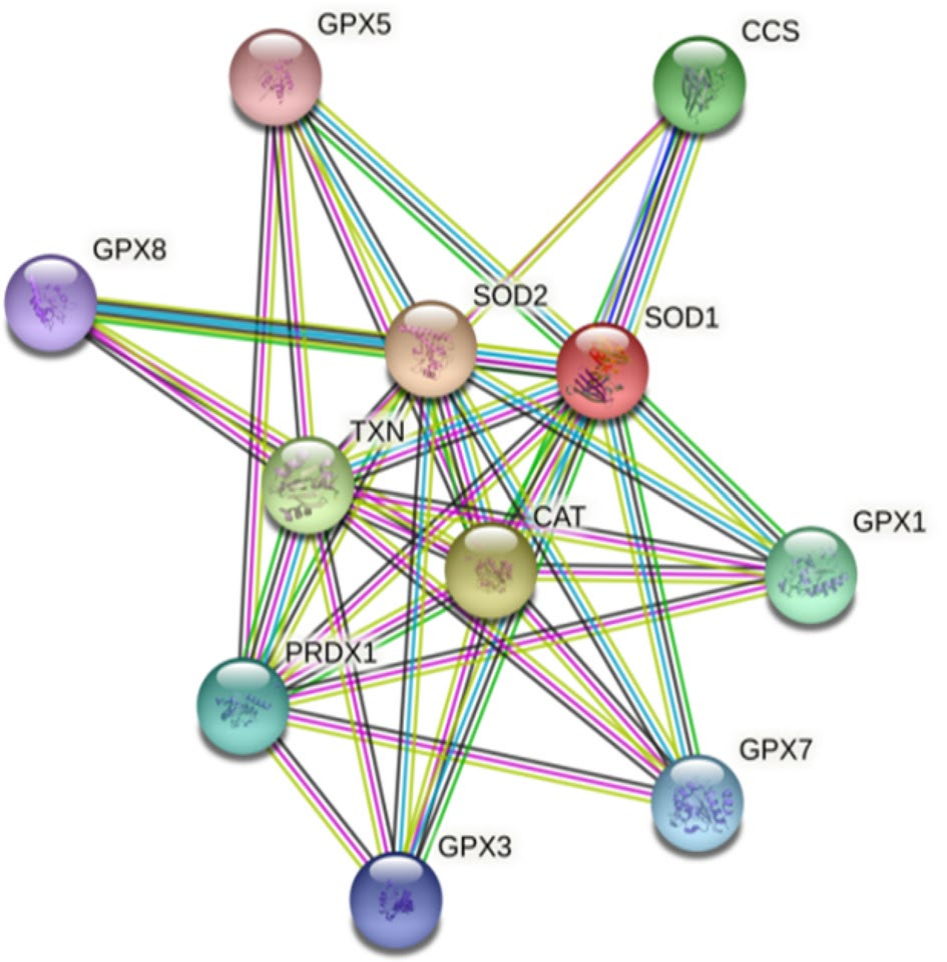
Predicted interacting partners of SOD1 and SOD2 genes.

The whole transcriptome gene expression profile in public databases, viz.: GEO2R does not provide clear indication about SOD1 and SOD2 dysregulation in RSA. It shows that SOD1 and SOD2 dysregulation is having a role in RSA. Therefore, to determine if genes SOD1 and SOD2 have any interaction with DEGs of RSA, this interactome was produced using STRING. It revealed 10 putative interactors viz*.*: CCS, SOD2, GPX1, GPX3, GPX5, GPX7, GPX8, CAT, PRDX1, TXN proteins of SOD1 while that for SOD2 were SOD1, SOD3, CAT, GPX1, GPX2, GPX3, GPX7, SIRT3 (Sirtuin-3 mitochondrial NAD-dependent deacetylase), FOXO3 (Forkhead box O), AKT1 (RAC-alpha serine/threonine-protein kinase) (Fig. 6).

**Figure 7:**
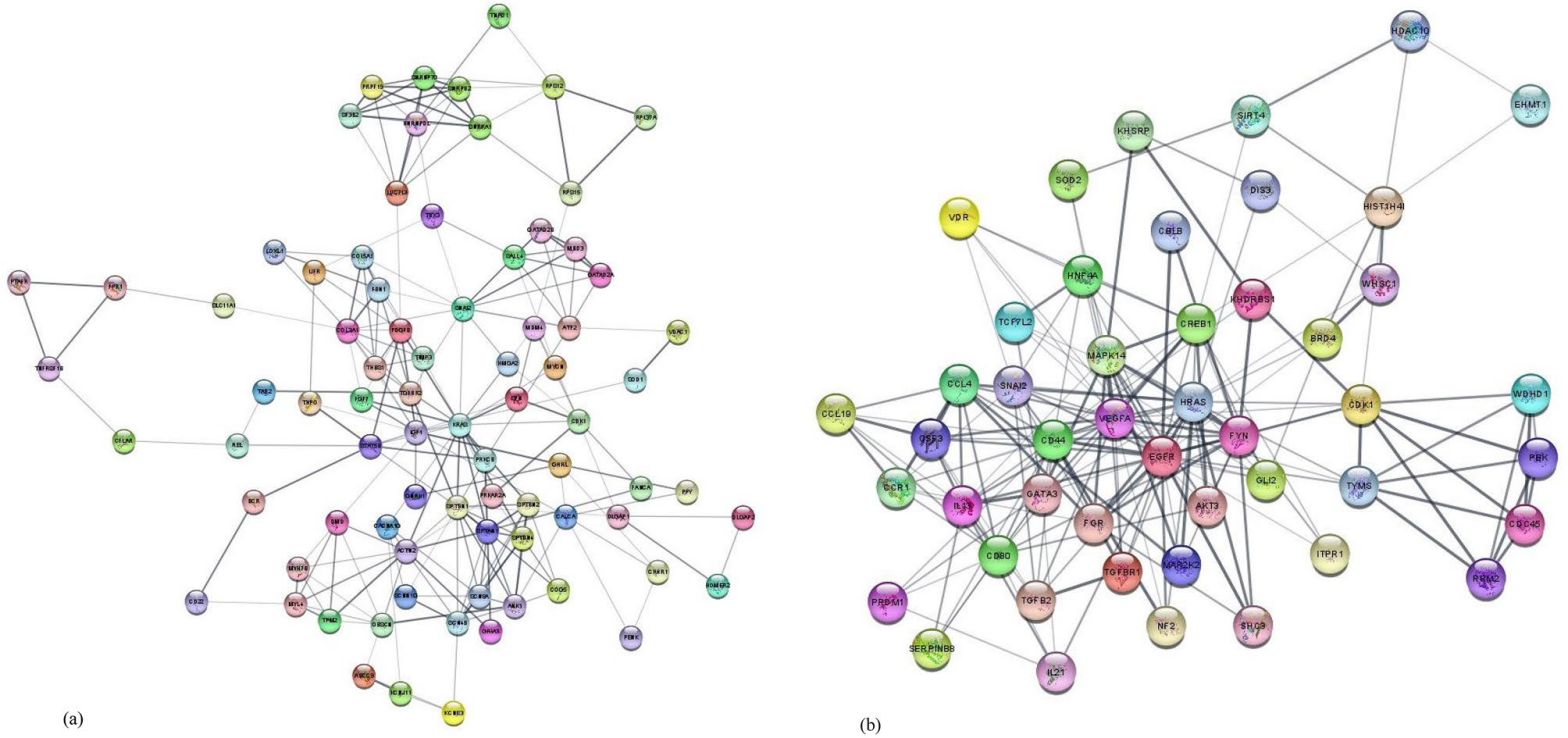
Differentially expressed genes protein–protein interaction with (**a**) SOD1 and (**b**) SOD2.

Dysregulated genes were integrated on Cytoscape software for biological function assessment and GO/KEGG pathway enrichment. MCODE analysis of DEGs on Cytoscape revealed two clusters of network showing interaction with SOD1 and SOD2 genes (Fig. 7a, b). Molecular function, biological process and cellular component domains were covered under GO. In case of SOD1 gene and its interacting partners, regulation of signal transduction and system processes was the most significant biological process (Table 2a). Dysregulated genes in RSA were a part of the intracellular cell component (Table 2b). Molecular function found to be most significant was protein/ enzyme binding (Table 2c). In case of SOD2 gene, cell proliferation, transcription and homeostasis were the significant biological processes for the dysregulated genes in RSA (Table 3a), while cellular component was intracellular (Table 3b) and protein binding was the significant molecular process (Table 3c). The results revealed that DEGs were markedly involved in MAPK, p53 signaling pathway, cell cycle pathway and chemokine signaling pathways (Tables 2d, 3d).

**Table 2:** Gene ontology and KEGG analysis of differentially expressed genes associated with SOD1 in recurrent spontaneous abortion: (a) biological process (b) cellular component (c) molecular function (d) KEGG pathway.

Term name	Description	Gene symbol	Gene count	P value
**(a)**				
GO:0023052	Signaling	THPO|PRPF19|SNRPA1|KRAS|TBX3|ABCC9|SPTBN4|LIFR|VDAC1|PRKAR2A|ANK1|FGF7|SOD1|GNRH1|STAT5B|REL|IGF1|BCR|COL3A1|PRKCB|HOMER2|KCNE3|CFLAR|DLGAP1|SCN4B|PENK|FBN1|PDGFB|CALCA|GHRL|SFN|SCN8A|SPTBN1|TGFBR2|CACNA1G|DMD|MDM4|TAB2|SPTAN1|PTAFR|TNFRSF1B|CDK1|SNAI2|CRHR1|DLGAP2|SPTBN2|HMGA2|ACTN2|OBSCN|PPY|FPR1|GRIA3|HNRNPDL	54	2.23E−13
GO:1901700	Response to oxygen-containing compound	SLC11A1|THBS1|ABCC9|LOXL1|ATF2|PRKAR2A|SOD1|GNRH1|STAT5B|IGF1|BCR|COL3A1|PRKCB|HOMER2|CFLAR|PENK|FBN1|PDGFB|KCNJ11|TGFBR2|TAB2|PTAFR|TNFRSF1B|CDK1|CRHR1|MBD3|ACTN2	26	6.40E−11
GO:0003008	System process	KRAS|TBX3|SPTBN4|VDAC1|PRKAR2A|TIMP3|SOD1|SCNN1G|IGF1|BCR|HOMER2|KCNE3|CFLAR|SCN4B|PENK|PDGFB|CALCA|GHRL|KCNJ11|SCN8A|MYL4|CACNA1G|DMD|PTAFR|TPM2|SNAI2|CRHR1|ACTN2|GRIA3	30	2.85E−10
GO:0035556	Intracellular signal transduction	PRPF19|KRAS|SPTBN4|PRKAR2A|FGF7|REL|IGF1|BCR|PRKCB|HOMER2|FBN1|PDGFB|CALCA|SFN|SPTBN1|DMD|MDM4|TAB2|SPTAN1|PTAFR|TNFRSF1B|CDK1|CRHR1|SPTBN2|ACTN2|FPR1	27	2.37E−09
GO:0044057	Regulation of system process	ABCC9|SPTBN4|SOD1|IGF1|KCNE3|SCN4B|PDGFB|CALCA|GHRL|KCNJ11|MYL4|CACNA1G|DMD|PTAFR|TNFRSF1B|CRHR1	17	2.50E−09
**(b)**				
GO:0032991	Protein-containing complex	SALL4|PRPF19|RPS12|SNRPB2|SNRPA1|THBS1|ABCC9|MYH7B|SPTBN4|LIFR|VDAC1|PRKAR2A|SOD1|REL|COG5|SCNN1G|IGF1|BCR|COL3A1|PRKCB|KCNE3|CFLAR|SF3B2|SCN4B|KCNJ11|SCN8A|MYL4|SPTBN1|TGFBR2|CACNA1G|GATAD2A|DMD|GATAD2B|COL5A1|SPTAN1|TNFRSF1B|TPM2|FANCA|CDK1|MBD3|RPL37A|LUC7L3|SPTBN2|HMGA2|RPS15|SNRNP70|GRIA3|HNRNPDL	48	1.53E−10
GO:0030141	Secretory granule	SLC11A1|THBS1|LOXL1|TIMP3|SOD1|IGF1|PENK|PDGFB|GHRL|KCNJ11|SPTAN1|PTAFR|TNFRSF1B|ACTN2|FPR1	15	1.70E−06
GO:0070013	Intracellular organelle lumen	SALL4|PRPF19|RPS12|SNRPB2|SNRPA1|TBX3|THBS1|SPTBN4|ATF2|VDAC1|TIMP3|SOD1|MYCN|STAT5B|REL|COG5|SCNN1G|IGF1|COL3A1|PRKCB|SF3B2|PENK|FBN1|PDGFB|GHRL|SPTBN1|GATAD2A|MDM4|TAB2|GATAD2B|COL5A1|SPTAN1|FANCA|CDK1|SNAI2|MBD3|LUC7L3|HMGA2|ACTN2|OBSCN|RPS15|SNRNP70|HNRNPDL	43	7.97E−06
**(c)**				
GO:0005488	Binding	CD22|THPO|SALL4|PRPF19|RPS12|SLC11A1|SNRPB2|SNRPA1|KRAS|TBX3|THBS1|ABCC9|LOXL1|MYH7B|SPTBN4|LIFR|ATF2|VDAC1|PRKAR2A|ANK1|TIMP3|FGF7|SOD1|GNRH1|MYCN|STAT5B|REL|SCNN1G|IGF1|BCR|COL3A1|PRKCB|HOMER2|KCNE3|CFLAR|DLGAP1|SF3B2|SCN4B|PENK|FBN1|PDGFB|CALCA|GHRL|TNPO1|SFN|KCNJ11|SCN8A|MYL4|SPTBN1|TGFBR2|CACNA1G|GATAD2A|DMD|MDM4|TAB2|GATAD2B|COL5A1|SPTAN1|PTAFR|TNFRSF1B|TPM2|CDK1|SNAI2|CRHR1|MBD3|RPL37A|LUC7L3|SPTBN2|HMGA2|ACTN2|OBSCN|PPY|RPS15|FPR1|SNRNP70|GRIA3|HNRNPDL	77	5.09E−12
GO:0044877	Protein-containing complex binding	CD22|SNRPB2|KRAS|THBS1|MYH7B|SPTBN4|ATF2|VDAC1|ANK1|IGF1|COL3A1|HOMER2|CFLAR|DLGAP1|FBN1|PDGFB|CALCA|MYL4|SPTBN1|TGFBR2|COL5A1|TPM2|HMGA2|ACTN2	24	6.81E−11
GO:0003779	Actin binding	MYH7B|SPTBN4|HOMER2|MYL4|SPTBN1|DMD|SPTAN1|TPM2|SPTBN2|ACTN2	10	1.36E−05
GO:0019899	Enzyme binding	CD22|SPTBN4|ATF2|VDAC1|PRKAR2A|ANK1|TIMP3|SOD1|MYCN|BCR|COL3A1|PRKCB|CFLAR|TNPO1|SFN|SPTBN1|TGFBR2|DMD|MDM4|PTAFR|TNFRSF1B|OBSCN	22	7.01E−05
**(d)**				
hsa04010	MAPK signaling pathway	KRAS|ATF2|FGF7|IGF1|PRKCB|PDGFB|TGFBR2|CACNA1G|TAB2	9	3.31E−06
hsa04115	p53 signaling pathway	THBS1|IGF1|SFN|MDM4|CDK1	5	1.55E−05
hsa05202	Transcriptional misregulation	MYCN|REL|IGF1|TGFBR2|HMGA2|GRIA3	6	8.50E−05
hsa04068	FoxO signaling pathway	KRAS|IGF1|HOMER2|TGFBR2	4	0.002

**Table 3:** Gene ontology and KEGG analysis of differentially expressed genes associated with SOD2 in recurrent spontaneous abortion. (a) Biological process (b) cellular component (c) molecular function (d) KEGG pathway.

Term name	Description	Gene symbol	Gene count	P value
**(a)**				
GO:0048519	Negative regulation of biological process	SIRT4|HDAC10|CSF3|MAPK14|MAP2K2|CREB1|GLI2|CD44|CDC45|HRAS|EHMT1|PBK|SOD2|VDR|VEGFA|HIST1H4I|CCL4	17	1.65E−21
GO:0006357	Regulation of transcription by RNA polymerase II	HDAC10|CSF3|MAPK14|BRD4|EGFR|HNF4A|KHDRBS1|TGFB2|PRDM1|TCF7L2|GATA3|WHSC1|SNAI2|KHSRP|CREB1|GLI2|HRAS|EHMT1|SOD2|VDR|VEGFA	20	6.62E−09
GO:0008285	Negative regulation of cell population proliferation	CD80|HNF4A|NF2|TGFB2|TGFBR1|GATA3|SNAI2|HRAS|SOD2|VDR	10	5.31E−06
GO:0048878	Chemical homeostasis	EGFR|CCR1|IL13|ITPR1|CCL19|HNF4A|TCF7L2|SOD2|VDR|VEGFA	10	2.90E−04
**(b)**				
GO:0070013	Intracellular organelle lumen	SIRT4|HDAC10|MAPK14|BRD4|CBLB|EGFR|ITPR1|HNF4A|KHDRBS1|TYMS|NF2|WDHD1|TGFB2|PRDM1|TCF7L2|FGR|DIS3|GATA3|WHSC1|CDK1|SNAI2|KHSRP|CREB1|GLI2|CDC45|HRAS|EHMT1|SOD2|VDR|VEGFA|HIST1H4I	31	3.84E−07
**(c)**				
GO:0005126	Cytokine receptor binding	CSF3|IL21|IL13|CCL19|TGFB2|TGFBR1|GATA3|VEGFA|CCL4	9	1.48E−08
GO:0005488	Binding	SIRT4|HDAC10|CSF3|MAPK14|MAP2K2|BRD4|AKT3|CBLB|CD80|IL21|EGFR|CCR1|IL13|ITPR1|CCL19|HNF4A|KHDRBS1|TYMS|NF2|FYN|RRM2|WDHD1|TGFB2|PRDM1|TCF7L2|FGR|TGFBR1|SHC3|DIS3|GATA3|WHSC1|CDK1|SNAI2|KHSRP|CREB1|GLI2|CD44|CDC45|HRAS|EHMT1|PBK|SOD2|VDR|VEGFA|HIST1H4I|CCL4	46	2.15E−08
GO:0003682	Chromatin binding	BRD4|EGFR|HNF4A|WDHD1|PRDM1|TCF7L2|WHSC1|CDK1|SNAI2|GLI2|CDC45	11	9.28E−08
GO:0004672	Protein kinase activity	MAPK14|MAP2K2|BRD4|AKT3|EGFR|FYN|FGR|TGFBR1|CDK1|PBK	10	8.86E−07
**(d)**				
hsa04926	Relaxin signaling pathway	MAPK14|MAP2K2|AKT3|EGFR|TGFBR1|SHC3|CREB1|HRAS|VEGFA	9	3.30E−11
hsa04664	Fc epsilon RI signaling pathway	MAPK14|MAP2K2|AKT3|IL13|FYN|HRAS	6	1.89E−08
hsa04218	Cellular senescence	MAPK14|MAP2K2|AKT3|ITPR1|TGFBR1|CDK1|HRAS	7	8.85E−08
hsa04062	Chemokine signaling pathway	AKT3|CCR1|CCL19|FGR|SHC3|HRAS|CCL4	7	3.61E−07
hsa04211	Longevity regulating pathway	AKT3|CREB1|HRAS|EHMT1|SOD2	5	2.67E−06

## Discussion

RSA has been a challenging and a complex condition with idiopathic etiology. The etiology is unexplained and poorly understood in most of the cases thus making it difficult for physicians to treat. Homeostatic balance of antioxidants and oxidants is imperative towards maintaining normal cellular physiological functions. ROS are important for several physiological and cell signaling functions but also play a major role in pathology of reproductive processes. Their overproduction leads to cellular damage and can contribute to pathogenesis of diseases by impeding normal physiological activities of the cell. Previous studies have suggested that release in excessive oxygen radicals can lead to pregnancy complications such as pre-eclampsia, spontaneous abortion and also reproductive associated diseases such as polycystic ovarian syndrome, endometriosis^29–31^. SODs require the presence of oxygen and metal ions cofactors for their function. Present in subcellular localization; Cu–Zn SOD in the cytoplasm and extracellularly and MnSOD in the mitochondria^32^.

Unsuccessful maintenance of pregnancy due to *C. trachomatis* infection places a major burden on women. The pathogen evades the upper genital tract causing scarring and occlusion of fallopian tube, pelvic inflammatory diseases and in extreme cases infertility. The host pathogen interaction involves an array of participating signaling molecules like cytokines and ROS. *C. trachomatis* induces the release of ROS causing series of events leading to cell damage which eventually progresses to chronic infection. Few studies have been conducted in *C. trachomatis-*associated OS in reproductive pathology. A study suggested *C. trachomatis* infection as a predominant factor in the pathogenesis of abortion as it increases production of ROS and thereby OS leading to unfavorable pregnancy outcomes^33^. A similar study reported lower antioxidant capacity in patients with tubal infertility *C. trachomatis-*positive women compared to fertile *C. trachomatis*-negative control^34^.

In the present study, mRNA expression of SOD1 and SOD2 genes was determined in *C. trachomatis*-infected RSA and control patients in urine by q-RT-PCR. Our data confirmed significant upregulation of SOD2 gene expression in Group Ia when compared to Group II (‘p’ > 0.05) whereas decreased expression was observed in Group Ib patients *versus* Group II indicating that its role is more prevalent in uninfected RSA patients. In case of SOD1, downregulated expression was observed in Group Ia when compared to Group II (‘p’ > 0.05) suggesting that SOD1 has a significant role in continuation of pregnancy by preventing the accumulation of ROS radicals as its expression was decreased in *C. trachomatis*-infected RSA patients as compared to controls. On comparison of Group Ib and Group II, SOD1 expression was higher in Group Ib patients implying that it has a greater role in infected recurrent aborters. A fold change of 0.67 and 1.79 was observed on comparing Group Ia and Ib for SOD1 and SOD2 gene respectively indicating the role of *C. trachomatis* infection in downregulating the expression of SOD1 in RSA patients.

A significant correlation was observed between SOD2 gene and GA in *C. trachomatis*-positive RSA patients while no significant correlation was observed between SOD1 gene and GA in *C. trachomatis*-positive RSA patients. Chlamydial load showed positive correlation with SOD2 while a negative correlation was observed with SOD1 gene suggesting that greater chlamydial load leads to decreased expression of SOD1 gene. Elevated levels of 8-OHdG and 8-isoprostane were observed in Group Ia when compared to controls (p < 0.05) indicating that these biomarkers might be involved in the underlying pathological mechanism adopted by *C. trachomatis* in inducing RSA.

Further, interactome map was constructed for SOD1 and SOD2. Potential interacting partners of these proteins were predicted by STRING database. For SOD1 the interacting proteins were: CCS, SOD2, GPX1, GPX3, GPX5, GPX7, GPX8, CAT, PRDX1, TXN while that for SOD2 were SOD1, SOD3, CAT, GPX1, GPX2, GPX3, GPX7, SIRT3 (Sirtuin-3 mitochondrial NAD-dependent deacetylase), FOXO3 (Forkhead box O), AKT1 (RAC-alpha serine/threonine-protein kinase). The common proteins for both SOD1 and SOD2 were GPX1, GPX3, GPX7 and CAT.

Several studies have implicated the association of the above mentioned interacting partners in regulation of OS in RSA and *C. trachomatis* infection suggesting their role together with SOD genes. For instance, PRDX2, one of the interacting partners of SOD genes was reported to have significantly lower expression in trophoblast cells of RSA patients. Subsequent to knockdown of PRDX2 gene the cellular ROS levels increased which led to proliferation and apoptosis^35^. In another study PRDX3 and PRDX4 have been shown to play pivotal role in implantation and normal placentation through their antioxidant activity. The family of peroxiredoxins plays important roles in neutralizing OS. Peroxiredoxins also interact with thioredoxins by maintaining its reduction status and further downregulating pro-apoptotic pathways^36^. FOXO proteins (another interacting partner of SOD genes) are key regulators in cell cycle progression, cell differentiation, DNA repair, apoptosis and cell differentiation^37^ and one of the most activated pathways during chlamydial infection^38^. In a study it has been demonstrated that decidualizing cells are resistant to oxidative cell death due to function of antioxidant genes particularly SOD2, thioredoxin, peroxiredoxin and if exposed to OS, it induces FOXO expression alleviating ROS mediated apoptosis^39^. Glutathione along with GPX plays a key role in determining the progression of *C. trachomatis* infection and its developmental cycle implying susceptibility to ROS^40^. These findings suggest that SOD genes with its interacting partners might be playing critical roles in pathology of *C. trachomatis* infection during implantation and pregnancy.

GO/KEGG analysis revealed that the DEGs were involved in biological and molecular processes such as protein binding, intracellular transduction signaling process, cell proliferation and transcription regulation. MAPK, cell cycle, chemokine and apoptotic pathways were involved for these DEGs found to be interacting with SOD1 and SOD2 genes. Genes enriched in the MAPK pathway were FGF7, IGF1, and KRAS which are known to be involved in RSA. These genes have a major role in migration, proliferation and invasion of cells during implantation*.* Similarly, the enriched genes of the p53 pathway were MDM4, CDK1 and IGF1 which have diverse functions in cell cycle progression and apoptosis. Therefore, dysregulation of these molecular pathways can result in pathogenesis of RSA. Higher levels of p53 were detected in placental villi resulting in apoptosis by mediating trophoblast infiltration and eventually spontaneous abortion^41,42^. These genes also play important role during *C. trachomatis* infection. *C. trachomatis* is highly dependent on MDM2-p53 interaction. Inhibition of the p53-MDM2 interaction disrupts intracellular development and interferes with the pathogen’s anti-apoptotic effect on host cells^43^. *C. trachomatis* manipulates the eukaryotic cell cycle by destabilizing CDK1 proteins^44^. In another study, it was shown that FGF7 regulates proliferation of endometrial cells via the MAPK pathway^45^.* C. trachomatis* has also been known to induce its infection by MAPK/ERK pathways by stimulating FGF and enhancing its infection and spread leading to apoptosis of host cells^46,47^. A finding suggests that the effect of *C. trachomatis* infection on trophoblast involves the chemokine signaling pathways leading to expression of innate immune receptors by the trophoblast and virulence factors secreted into the trophoblast by the bacteria^48^. This type of cross talk as in the case of infection induced inflammation could be responsible for *C. trachomatis*-induced spontaneous abortion. All these pathways finally diverge to major cellular processes such as cell cycle, apoptosis, DNA repair and ROS detoxification involving the enriched genes found in our study. It can be further concluded that antioxidant genes are important part of signaling pathways interlinked with a network of other genes leading to ROS detoxification and associated cell physiological pathways.

## Methods

### Ethical approval, enrolment of patients and collection of clinical sample

Ethical approval for the study was obtained from the Institutional Ethics Committee, VMMC and Safdarjung Hospital, New Delhi, India (IEC/VMMC/SJH/Project/2019/08/42). Thereafter, the present case–control study was undertaken in 130 non-pregnant women attending Department of Obstetrics and Gynecology, Safdarjung Hospital, New Delhi, India having a history of RSA in the first trimester (Group I) while 130 age-matched asymptomatic non-pregnant women having history of two successful deliveries served as controls (Group II). Written consent was obtained from each patient prior to collection of samples.

Recurrent aborters with recent antibiotic therapy, chromosomal abnormalities, autoimmune diseases such as diabetes, metabolic disorders like thyroid and anatomical factors like endometriosis and uterine anomalies were excluded from the study. Patients with previous history of genitourinary tract infection, HIV-positivity, VDRL-positivity, TORCH (*Toxoplasma gondii, Rubella, Cytomegalovirus, Herpes simplex virus*) were not taken under study. *Mycoplasma genitalium, Neisseria gonorrhoeae, Trichomonas vaginalis, Ureaplasma urealyticum, Ureaplasma parvum, Herpes simplex virus ½* were detected in urine by Fast Track Diagnostics STD9 real time PCR kit (Siemens Healthcare, Germany) according to manufacturer’s protocol. The amplification was detected in DNA samples by fluorescent reporter dye probes specific to each pathogen with an internal control. Obstetric history information like gravidity, parity, abortion history, and last menstrual period was recorded in questionnaire from all patients enrolled in the study. All experiments were performed in accordance with relevant guidelines and regulations (including informed consent from all participants).

Urine (15–20 ml) was collected from patients enrolled in Groups I and II. Transportation of samples to the laboratory was done on ice. Centrifugation of samples was done at × 1700 rpm for 30 min at 4 °C, the supernatant was discarded and the pellet was stored for further use at − 80 °C.

### *Chlamydia trachomatis* detection in urine

DNA was isolated from urine after dissolving the urine pellet in lysis buffer. Thereafter, DNA was precipitated in propanol and eluted in 50 µl nuclease-free water^23^. Detection of *C. trachomatis* by conventional PCR was performed in both groups by amplifying chlamydial endogenous cryptic plasmid gene and MOMP gene of 200 and 537 bp respectively. MOMP (5′ TAT ACA AAA ATG GCT CTC TGC TTT AT 3′ and 5′ CCC ATT TGG AAT TCT TTA TTC ACA TC 3′) and plasmid (5′ CTA GGC GTT TGT ACT CCG TCA 3′ and 5′ TCC TCA GGA GTT TAT GCA CT 3′) primer pair sequences were obtained from the published literature^24,25^ and these primers were commercially synthesized (Biolink, New Delhi, India). Negative control or no template control and positive control were set up using *C. trachomatis* DNA (Amplirun Vircell, Spain). Amplification reactions were performed as described earlier^26^.

### Detection of *Chlamydia trachomatis* load in urine by quantitative real-time PCR

Quantitative real-time PCR (q-PCR) assay was performed to detect the chlamydial load in *C. trachomatis-*positive urine samples. Serial dilutions (10,000, 1000, 100, 10, 0.1 μl) of *C. trachomatis* DNA control (*Vircell Microbiologist, Granada, Spain*) were prepared as per the manufacturer’s instructions. 10 μl of SYBR green master mix, 1 μl of plasmid gene (200 bp) forward primer and 1 μl of reverse primer, 5 μl of Vircell *C. trachomatis* diluted DNA of each concentration and 3 μl of nuclease-free water were mixed to make up a final volume of 20 μl. A standard curve was drawn. By using this standard curve, the chlamydial load was calculated in each sample.

### Estimation of 8-OHdG and 8-isolprostane in urine by ELISA

Urine 8-OHdG and 8-isolprostane concentration was determined by a commercial EIA kit (Fine Biotech, Co., Ltd, China) as per the manufacturer’s guidelines. Briefly, 50 μl standard and samples were added to the wells. Immediately, 50 μl Biotin labeled antibody was added and incubated for 45 min at 37 °C. After washing, 100 μl HRP-Streptavidin conjugate working solution was added into each well and incubated for 30 min at 37 °C.

Subsequently, the plate was washed, TMB substrate was added to each well, and the plate incubated for 20 min at 37 °C. Stop solution was added and the plate was read in an ELISA reader (Titertek, Finland) at 450 nm.

### Quantitative analysis of SOD1 and SOD2 genes by Real-Time PCR

RNA was isolated by Trizol (Invitrogen, USA) method in Group I and II patients according to the manufacturer’s guidelines. Briefly, 15–20 ml urine collected was pelleted and thereafter homogenized with 1 ml Trizol and 200 µl chloroform. The supernatant was separated and centrifuged. After addition of 75% ethanol to the supernatant, it was re-centrifuged and the pellet was air-dried. 30 μl of nuclease-free water was used for eluting RNA and the isolated RNA was kept at − 80 °C for future use. Expression of SOD1 and SOD2 genes was studied by real-time PCR assay performed on Step One Plus (Applied Biosystems, USA). A concentration of about 1.5-mg/ml of cDNA was used for performing real time PCR. A 20 μl reaction was set up for the experiment. In brief, 10 μl Sybr Green master-mix, 1 μl of primer, 4 μl of cDNA, and 5 μl of sterile water were added to make up the total volume. Primers sequences (SOD1-5′CGAGCAGAAGGAAAGTAATG3′ and 5′TAGCAGGATAACAGATGAGT3′; SOD2-5′AGTTCAATGGTGGTGGTCATA3′ and 5′CAATCCCCAGCAGTGGAATAA3′) were obtained from published literature and commercially synthesized (Biolink, New Delhi, India)^27^. Glyceraldehyde 3-Phosphate Dehydrogenase (GAPDH) was used as an internal control. The mean threshold cycle (Ct) value was calculated for the target genes and endogenous control and gene expression study was done by relative quantification.

### Statistical evaluation

Graph pad Prism (version 8.0) was used for statistical evaluation. Fold change was calculated using 2^− ΔΔCT^ calculation and analyzed by Mann Whitney test/non-parametric test for comparing mean between two groups. The correlation between SOD genes and GA was analyzed using Spearman’s rank correlation test. ‘p’ value < 0.05 was considered to be significant.

### PPI with RSA differentially expressed genes (DEGs)

To search for potential interacting partners of SOD1 and SOD2 gene, the Search Tool for the Retrieval of Interacting Genes (STRING; http://string-db.org/) was applied. Functional enrichment analysis by KEGG was studied to decipher the reactome pathways^28^. Four gene expression profiles involved in RSA were retrieved from DEGs using Gene Expression Omnibus (GEO2R). PPI network was generated using the STRING app in Cytoscape. Based on DEGs of four datasets, interaction of SOD1 and SOD2 with DEGs and hub genes was evaluated. After the network was clustered into several groups, Molecular Complex Detection (MCODE; apps.cytoscape.org/apps/mcode) was used to identify the important clusters.

## Data Availability

The datasets generated used and/ or analysed during the current study available from the corresponding author on reasonable request. All data analysed during this study is included in the article.
